# Screen Time and Chronic Pain Health: Mendelian Randomization Study

**DOI:** 10.2196/78233

**Published:** 2026-02-09

**Authors:** Jiahui Jiang, Chunyan Pu, Jiarui Cai, Chuan Yu, Zhenmi Liu, Chenghan Xiao

**Affiliations:** 1 Department of Maternal and Child Health West China School of Public Health and West China Fourth Hospital Sichuan University Chengdu, Sichuan China; 2 The Medical Laboratory Center, Hainan General Hospital, Hainan Medical University Hainan Hospital Haikou, Hainan China

**Keywords:** causality, chronic pain, medical genetics, multisite chronic pain, screen time

## Abstract

**Background:**

The rapid proliferation of electronic devices has increased screen time, raising concerns about its potential health effects, including chronic pain. However, existing studies have limitations in scope and causal inference, with inconsistent findings and a lack of exploration of potential biological mechanisms.

**Objective:**

The objective of our study was to investigate the causal associations and potential shared biological mechanisms between different forms of screen time and various chronic pain phenotypes.

**Methods:**

Leveraging genome-wide association study data, we investigated the association and potential shared biological mechanisms between screen time (time spent watching television, time spent using computer, and length of mobile phone use) and chronic pain phenotypes (including multisite chronic pain [MCP], back, knee, neck or shoulder, hip pain, and headaches). Two-sample Mendelian randomization (MR), reverse MR and multivariable Mendelian randomization (MVMR) analysis were performed to examine associations between screen time and chronic pain. Summary data–based Mendelian randomization (SMR), transcriptome-wide association study (TWAS), and colocalization analysis were used to identify the shared genes and potential biological mechanism.

**Results:**

MR analysis revealed that time spent watching television and length of mobile phone use were positively associated with several types of chronic pain, while time spent using computer showed a negative association. Specifically, time spent watching television was positively associated with the risk of MCP (*P*=1.05×10^–31^; odds ratio [OR] 1.61, 95% CI 1.49-1.74), back pain (*P*=2.41×10^–8^; OR 1.14, 95% CI 1.09-1.19), knee pain (*P*=7.10×10^–6^; OR 1.09, 95% CI 1.05-1.13), neck or shoulder pain, and hip pain. Length of mobile phone use was positively associated with the risk of MCP (*P*=2.15×10^–5^; OR 1.22, 95% CI 1.11-1.34), headaches, and neck or shoulder pain. However, time spent using computer was negatively associated with the risk of MCP (*P*<.001; OR 0.83, 95% CI 0.75-0.92), back pain, and knee pain. The reverse MR results showed that MCP was positively associated with time spent watching television (*P*=4.8×10^–7^; OR 1.27, 95% CI 1.16-1.4) and length of mobile phone use (*P*=3.38×10^–5^; OR 1.29, 95% CI 1.14-1.45), while the association with time spent using computer (*P*=.61; OR 0.97, 95% CI 0.87-1.09) was not statistically significant. The MVMR results failed to meet the criterion that all conditional F-statistics exceed 10. Integrative 3 analysis methods identified overlapping genes, with *CEP170* emerging as a key gene consistently supported by SMR, TWAS, and colocalization analysis in the relationship between time spent using computer and MCP.

**Conclusions:**

Our findings demonstrate an association between screen time and various aspects of chronic pain. The *CEP170* gene might contribute to the shared biological mechanism between time spent using computer and MCP risk. However, due to the absence of robust MVMR results, the potential influence of confounding factors cannot be ruled out.

## Introduction

With the widespread popularity of electronic devices, the screen time spent by people on phones, computers, and televisions has increased significantly every day. Recently, an increasing number of studies have shown that excessive screen time may bring a series of health risks [1,2]. A study on children and adolescents has shown that long-term screen time exposure is closely related to obesity, restricted cognitive and language development, decreased academic performance, and altered sleep patterns [3,4]. Studies on adults have found that excessive screen time is associated with reduced physical activity and increased BMI [5]. Moreover, studies have found that the incidence of headaches increases with increased screen time, with similar trends observed in both adolescents and adults [6-9]. Research has also shown that prolonged computer use can contribute to pain in several areas, such as the neck, shoulders, and back [10].

While some studies have explored the relationship between screen time and chronic pain, the current evidence is still limited. An important limitation is that existing studies have shortcomings in assessment scope and underlying relationship to fully reveal the association between the two. We found that most studies have focused on the use of a single type of screen time. One study only pointed out that prolonged computer use can cause pain in multiple areas [10], but ignored the effects of different types of screen time. Moreover, most studies on screen time and chronic pain tend to focus on specific body regions, such as lower back pain or headaches [11,12], while overlooking the impact of screen time on multisite chronic pain (MCP) and the combined effects of different types of chronic pain. In addition, the results of existing studies are inconsistent, with some studies finding an association between screen time and chronic pain [13], while others fail to observe a significant association [14]. This inconsistency may be caused by confounding bias or a weaker ability to infer causality.

Another important limitation in the current research concerns the mechanisms associated with screen time and chronic pain. Some studies have suggested that the brightness or light wave frequency of screens may trigger migraine attacks, while prolonged exposure to screens may lower the threshold for migraines [15-17], making them more likely to be triggered by other factors. However, this explanation lacks a detailed understanding of the biological mechanisms involved. To date, the underlying biological mechanisms linking screen time and chronic pain have remained largely unexplored, highlighting the need for future research to address this issue and explore whether different devices cause chronic pain through common or different mechanisms.

The genome-wide association studies (GWASs) of MCP [18], the development of genetic epidemiology, and multiomics integration analysis methods provide new opportunities to address these limitations [19,20]. Two-sample Mendelian randomization (MR) analysis can be used to examine the potential relationship between screen time and chronic pain. MR analysis uses genetic variants significantly associated with exposure as instrumental variables (IVs) to infer causality, thereby minimizing confusion and reverse causality [21]. In addition, summary data–based Mendelian randomization (SMR) and transcriptome-wide association study (TWAS) analysis can help identify overlapping genes between the two. In addition, colocalization analysis can assess whether these associations share causal genetic variation, thereby strengthening the evidence for shared biological mechanisms. By using multiple genetic epidemiological methods, we can explore the biological relationship between screen time and chronic pain.

In this study, we aimed to address these limitations. First, we examined whether screen time was associated with MCP and chronic pain in 5 other body parts. Next, we identified overlapping genes through genetic epidemiological methods approach and explored their potentially shared biological mechanism, aiming to provide new insights into the complexity of chronic pain.

## Methods

### Study Overview

**Figure 1:**
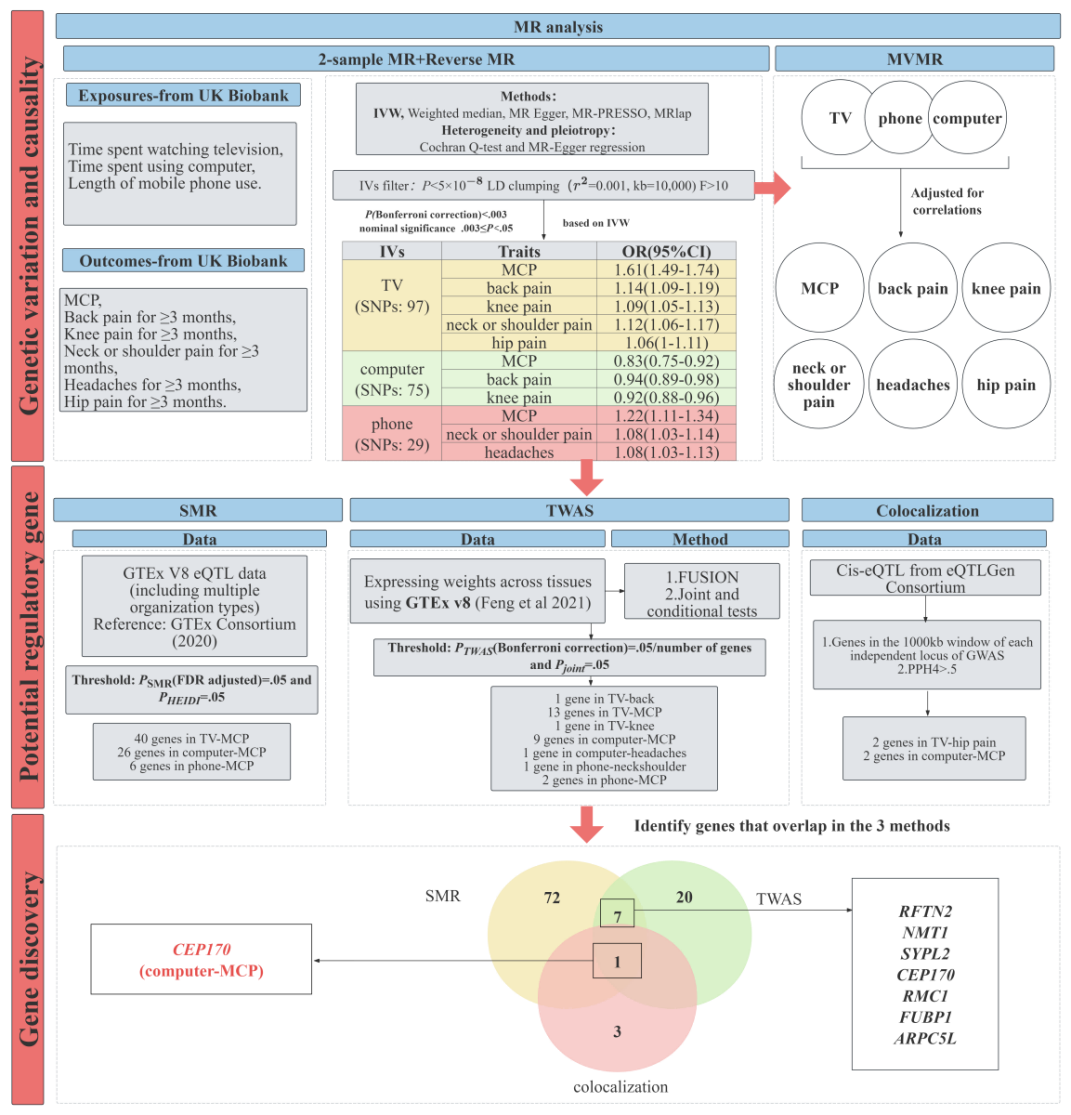
Flowchart of the overall study design. eQTL: expression Quantitative Trait Locus; FDR: false discovery rate; GTEx: Genotype-Tissue Expression; GWAS: genome-wide association study; IV: instrumental variable; IVW: inverse variance weighted; LD: linkage disequilibrium; MCP: multisite chronic pain; MR: Mendelian randomization; MVMR: multivariate Mendelian randomization; OR: odds ratio; SMR: summary data–based Mendelian randomization analysis; SNP: single-nucleotide polymorphism; TWAS: transcriptome-wide association study; TV: television.

As shown in Figure 1, this study follows a 3-stage approach. In Phase 1, we performed a 2-sample MR analysis and reverse MR to investigate the phenotypic association between screen time and chronic pain. Considering the mutual influence among multiple exposures, we conducted a multivariate Mendelian randomization (MVMR) analysis. In Phase 2, we first performed SMR analysis combined with expression Quantitative Trait Locus (eQTL) summary datasets to explore associations between gene expression and phenotypic traits. We then performed TWAS analysis to identify gene expression associated with screen time and chronic pain, and assessed whether the same genetic variants were shared through colocalization analysis, providing stronger genetic evidence. In Phase 3, we further integrated the results of SMR, TWAS, and colocalization analysis. We aimed to identify genes that were consistently supported across these methods. The overlapping genes provide more robust evidence of shared biological mechanisms in the association between screen time and chronic pain.

### Data Sources for Exposures, Outcomes, and eQTL Summary Data

#### Exposures

The exposure phenotype in this study was screen time, which included time spent watching television, time spent using computer, and length of mobile phone use. These phenotypes were derived from the UK Biobank touchscreen questionnaire. The question for time spent using computer explicitly stated, “Do not include using a computer at work,” whereas the questions for television watching and mobile phone use captured overall daily use. We provided detailed data sources about screen time in Multimedia Appendix 1. To address the issue of weak instrument bias, a genome-wide significance threshold of *P*<5×10^–8^ was used as the default criterion for identifying single-nucleotide polymorphisms (SNPs); simultaneously, to optimize the results and eliminate SNPs in strong linkage disequilibrium (LD), an LD clustering method was adopted, with thresholds of *r*^2^=0.001 and kilobase, kb=10,000 to ensure accurate and precise clustering. *r*^2^ is the squared correlation coefficient between 2 SNPs’ allele counts, used to quantify LD. In the 2-sample MR analysis, we selected 112, 82, and 32 independently significant SNPs located on the autosomes as IVs of time spent watching television, time spent using computer, and length of mobile phone use, respectively. Details of these SNPs are presented in Multimedia Appendices 2-4.

#### Outcomes

The outcomes included 6 chronic pain phenotypes, namely MCP, back pain for ≥3 months, knee pain for ≥3 months, neck or shoulder pain for ≥3 months, headaches for ≥3 months, and hip pain for ≥3 months. Among them, MCP is by far the largest GWAS summary statistic for all chronic pain phenotypes, involving 387,649 people from the UK Biobank [18]. Data for the remaining 5 types of chronic pain came from the Integrative Epidemiology Unit (IEU) OpenGWAS, which covered a total of about 9,851,867 SNPs in both male and female individuals [22]. Multimedia Appendix 1 presents the sources of these outcome data. The participants were all European populations to reduce bias caused by population heterogeneity. In reverse MR analysis, Multimedia Appendix 5 shows that 31 independently significant SNPs located on the autosomes as IVs of MCP.

#### eQTL Data

The Genotype-Tissue Expression (GTEx) project was established to characterize the genetic influence on transcriptomic variation across human tissues and to link regulatory mechanisms to traits and diseases. GTEx v8 provides 15,201 RNA-sequencing samples from 49 tissues [23], collected from 838 postmortem donors. The dataset comprehensively characterizes cis- and trans-eQTLs, revealing regulatory associations for nearly all genes and offering insights into allele heterogeneity, pleiotropy, and tissue-specific genetic effects. For SMR analysis, we used 8 eQTL summary data from GTEx (v8): Adipose Subcutaneous, Adipose Visceral Omentum, Brain Cerebellum, Brain Cortex, Brain Spinal cord cervical c-1, Muscle Skeletal, Nerve Tibial, and Whole Blood [23]. This is a set of cis-eQTL summary data for 8 human tissues from GTEx v8.

For colocalization analysis, we additionally used eQTL data from the eQTLGen Consortium [24]. eQTLGen provides a larger scale of eQTL data than GTEx, which can improve the confidence of colocalization analysis.

### Statistical Analysis

#### 2-Sample MR Analysis

2-sample MR analysis was conducted for exposure and outcome. The strength of IVs was assessed using the F-statistics: *F* = *R*^2^× (*N* – 2)/(1 – *R*^2^), where *R*^2^ represents the proportion of variation in the exposure variable explained by IVs [21]. The calculation of *R*^2^ involved multiplying beta (the estimated genetic association of each SNP with the trait) by minor allele frequency (MAF), using the formula 2 × MAF (1 – MAF) β^2^. An F-statistic >10 indicates a robust instrument [21]. IVs with F-statistics below 10 were considered weak instruments and excluded from analysis. We applied inverse variance weighted (IVW) regression as the primary method to estimate the effects of screen time on chronic pain [25]. In order to assess the robustness of the results, we also performed different sensitivity analyses. First, we complement the IVW MR with the weighted median, MR-Egger methods. The weighted median method can robustly estimate the causal relationship even when less than 50% of the genetic variants are invalid IVs [26]. The MR-Egger method includes an intercept term to account for directional pleiotropy [27]. Pleiotropy means that SNPs not only affect outcomes through exposures but also directly influence outcomes through other independent pathways. However, the slope of the MR-Egger regression provides valid MR estimates in the presence of horizontal pleiotropy [27,28]. It should be noted that when the number of SNPs is small, the statistical ability of MR-Egger is limited, making its results less reliable [27]. Second, we used Mendelian Randomization Pleiotropy Residual Sum and Outlier (MR-PRESSO) to detect the presence of outliers and to reassess the effect after removing the detected outlier SNPs [29]. Finally, we performed a leave-one-out analysis to reevaluate the IVW effect by excluding SNPs one by one [30]. To investigate potential bias due to sample overlap between screen time and chronic pain, we further conducted the MRlap method [31]. We examined the *P_difference_* of MRlap results, which is the *P* value used to test for differences between the observed (uncorrected) and corrected effects. We used the Bonferroni correction to account for multiple tests in the IVW result. An association was considered significant if the *P* value in the primary analysis was below 2.778×10^–3^ (.05/18) and the direction of effect estimates remained consistent across all methods. Correspondingly, suggestive evidence was considered if the *P* value for the IVW result was between .003 and .05.

To ensure methodological rigor and robustness, we also conducted a reverse MR, which is similar to the approach of 2-sample MR.

#### MVMR Analysis

To clarify the independent effects of each type of screen time on chronic pain, we performed MVMR analysis. The MVMR takes into account the combined effects of multiple related exposures, thereby adjusting for potential confounders and providing a more precise estimate of the independent contribution of each exposure. In the MVMR analysis, 3 phenotypes related to screen time exposures (time spent watching television, time spent using computer, and length of mobile phone use) were used as common exposure variables, and SNPs with *P*<5×10^–8^ genomic significance were used as IVs. At the same time, SNPs in strong LD with exposure variables were removed through LD clustering (*r*^2^=0.001, kb=10,000). Next, we merged the SNPs of 3 exposures and deleted the duplicate SNPs. To ensure validity, only SNPs present in the result data were retained and any SNPs that did not match were excluded. The IVW method was used for main effect estimation. We evaluated the strength of the IVs using the conditional F-statistics, which measure the strength of each exposure conditional on the others in the model. If the conditional F-statistics for all exposures exceeded the rule-of-thumb value of 10, the IVs were considered adequately strong for the purposes of MVMR [32].

#### SMR Analysis

We performed SMR analysis to explore whether gene expression is causally linked to both screen time and chronic pain [20], thereby identifying potential functional genes involved in the relationship. SMR integrates GWAS and eQTL data, enabling the investigation of associations between gene expression and phenotypic traits. In the SMR analysis, exposures and outcomes were analyzed separately with each eQTL summary data to assess whether gene expression is linked to both screen time and chronic pain. During the analysis, SNPs significantly associated with gene expression in cis-eQTL summary data were used as IVs, as they serve as genetic proxies for gene expression levels. These SNPs were selected based on their genome-wide significant association with gene expression in eQTL summary data. False discovery rate (FDR) correction was applied to control the FDR of multiple tests. Additionally, to verify whether the observed associations could be caused by a single causal variable, we evaluated the association results using the heterogeneity in dependent instruments (HEIDI) test implemented in the SMR tool, retaining only probes with *P_HEIDI_* values indicating low heterogeneity [33]. Ultimately, the SMR analysis results for screen time and chronic pain were integrated to identify overlapping genes and further explore their potential roles in the association between screen time and chronic pain.

#### TWAS Analysis

We conducted TWAS to identify genes whose expression may mediate the relationship between screen time and chronic pain. GWAS datasets were obtained from IEU OpenGWAS, and using the FUSION tool developed by Gusev et al [34], we performed single-trait TWAS using the 3 cross-tissue weights for cross-tissue features generated through sparse canonical correlation analysis (sCCA) on GTEx v8 gene expression (including sCCA1, sCCA2, and sCCA3) to identify regulatory genes that may be involved in chronic pain pathways [19]. To control for type I error rates, Bonferroni correction was applied to each exposure and result to interpret multiple tests, setting the significance level for each trait or tissue to *P*=.05/number of genes (adjusted for the number of genes on each chromosome in the 3 GTEx v8 weights). We then extracted significant GWAS of exposure and outcome, performed joint and conditional tests on loci with multiple related features to assess whether loci contain signals independent of expression. Finally, we sought overlapping genes between exposures and outcomes in joint tests.

#### Colocalization Analysis

To further verify the reliability of the SMR analysis results and determine whether screen time and pain are driven by the same genetic variation, we used colocalization analysis. First, we determined all independent signals of GWAS across the genome through LD independence analysis. Genes within a 1000-kb window of each independent locus were subjected to colocalization analysis [35]. Within these windows, we combined the corresponding genes with eQTL data, which is cis-eQTL data in SMR format, and then conducted colocalization analysis. We performed colocalization analysis using the default priors of *P_1_*=1×10^–4^, *P_2_*=1×10^–4^, and *P_12_*=1×10^–5^ [36]. Colocalization analysis assesses whether 2 traits may be driven by the same causal variant by estimating the joint posterior probability (PP) of GWAS and eQTL signals at the same locus. This method assumes a maximum of one causal variant per trait in a gene region and uses approximate Bayesian factor calculations to derive the PP for 4 mutually exclusive hypotheses (H_0_-H_4_), representing all possible association configurations between 2 traits: (1) H_0_: neither trait has a genetic association in the region; (2) H_1_ or H_2_: only trait 1 or trait 2 has a genetic association in the region; (3) H_3_: both traits are associated, but with different causal variants; and (4) H_4_: both traits are associated and share a single causal variant. The PP of each configuration is denoted as PPH_0_, PPH_1_, PPH_2_, PPH_3_, and PPH_4_, respectively [37]. We used PPH_4_ to characterize the possibility of colocalization. The probability value of .5< PPH_b_ <.8 suggests a moderate support for colocalization, whereas PPH_4_≥.8 indicates a strong support for colocalization, indicating that the 2 signals share a causal variant at this locus [38]. Using eQTL data, colocalization analysis was conducted separately for exposure and outcome traits, and genes showing evidence of colocalization were subsequently overlapped at the gene level.

Finally, to ensure the robustness of the analysis results, we further screened and consolidated the results of 3 analysis methods. We compared the colocalized genes with the overlapping genes identified in SMR and TWAS analysis to enhance the understanding of the potential biological mechanisms of these genes in the relationship between screen time and chronic pain.

### Ethical Considerations

All data used in this study were deidentified publicly available data; therefore, no ethical approval was required for this study. All original studies received ethical approval from their respective institutional review boards, and all participants provided informed consent. The data used were anonymized to ensure privacy and confidentiality. No compensation was provided to participants. Additionally, this study does not include any identifiable images or figures.

## Results

### The Putative Association Between Screen Time and Chronic Pain

**Figure 2:**
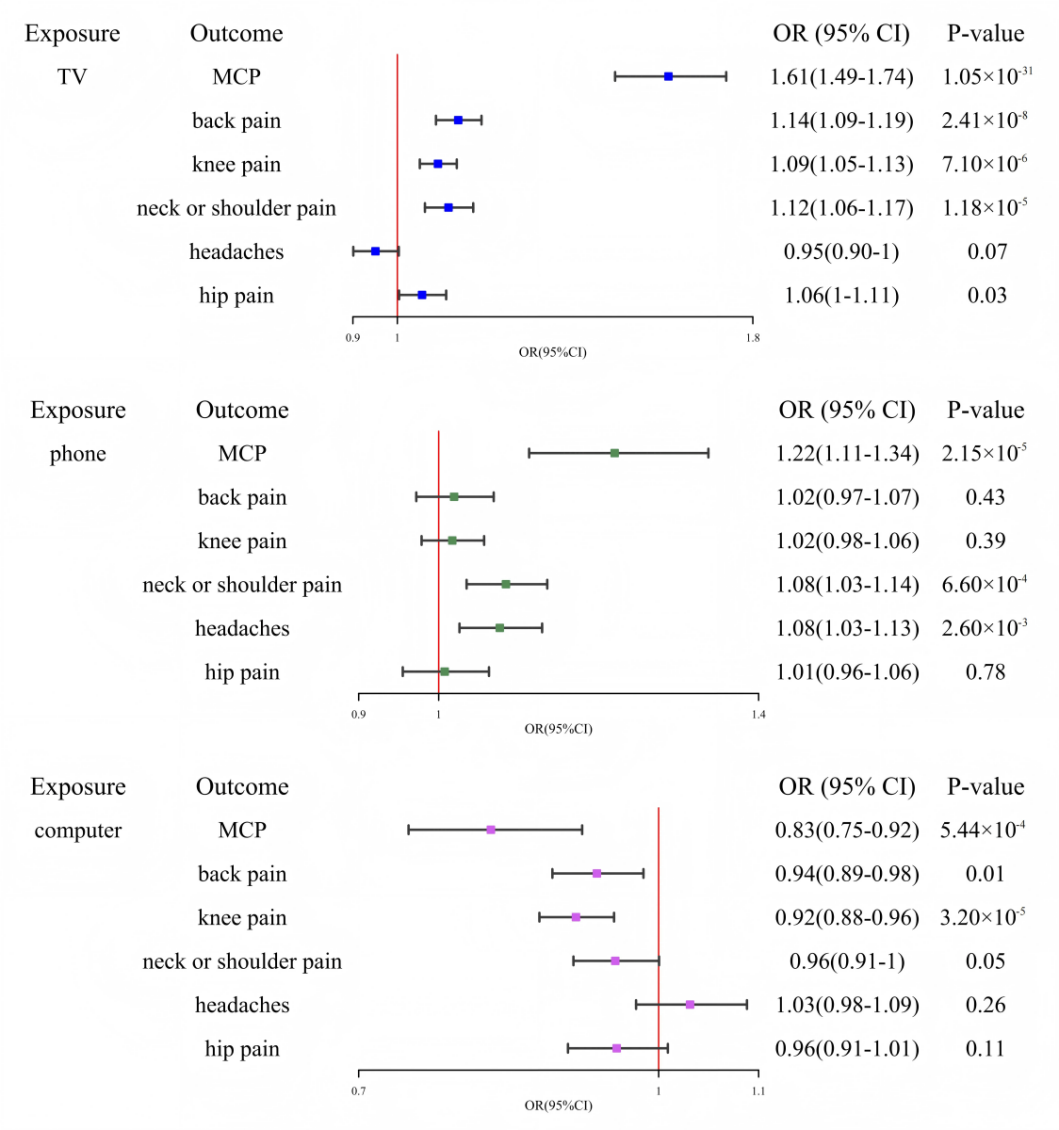
Mendelian randomization analysis of screen time and chronic pain based on the inverse variance weighted. MCP: multisite chronic pain; OR: odds ratio; TV: television.

The F-statistics of IVs are all greater than 10 in Multimedia Appendices 2-4, indicating that the IVs are relatively strong. We used a 2-sample MR method to make causal inference, primarily relying on the IVW method. Figure 2 shows that the 2-sample MR results of screen time and chronic pain based on the IVW. The box in Figure 2 indicates the point estimate of the causal effect, and the error bars represent the 95% CI. Multimedia Appendix 6 showed that time spent watching television and length of mobile phone use were positively associated with chronic pain, while time spent using computer was negatively associated. Our IVW results suggested significant positive associations of time spent watching television with MCP (*P*=1.05×10^–31^; odds ratio [OR] 1.61, 95% CI 1.49-1.74), back pain (*P*=2.41×10^–8^; OR 1.14, 95% CI 1.09-1.19), knee pain (*P*=7.10×10^–6^; OR 1.09, 95% CI 1.05-1.13), and neck or shoulder pain (*P*=1.18×10^–5^; OR 1.12, 95% CI 1.06-1.17). The association between time spent watching television and hip pain (*P*=.03; OR 1.06, 95% CI 1-1.11) reached nominal significance. It is notable that even though there is horizontal pleiotropy between time spent watching television and back pain (*P_MR-Egger intercept_*=.02), it is significant after MR-Egger correction (*P_MR-Egger slope_*=4.20×10^–4^). Similarly, our IVW results indicated significant positive associations of length of mobile phone use with MCP (*P*=2.15×10^–5^; OR 1.22, 95% CI 1.11-1.34), headaches (*P*=.003; OR 1.08, 95% CI 1.03-1.13), and neck or shoulder pain (*P*＜.001; OR 1.08, 95% CI 1.03-1.14). However, IVW results suggested significant negative associations of time spent using computer with MCP (*P*＜.001; OR 0.83, 95% CI 0.75-0.92), and knee pain (*P*=3.20×10^–5^; OR 0.92, 95% CI 0.88-0.96). The association between time spent using computer and back pain (*P*=.01; OR 0.94, 95% CI 0.89-0.98) reached nominal significance. For the significant associations, concordant estimates were basically suggested by weighted median, MR-Egger, MR-PRESSO, and MRlap.

MRlap-corrected results in Multimedia Appendix 6 showed that time spent watching television significantly increased the risk of MCP (*P*_corrected_=3.57×10^–30^; OR_corrected_=1.53, 95% CI 1.43-1.65), back pain (*P*_corrected_=8.46×10^–6^; OR_corrected_=1.27, 95% CI 1.14-1.41), knee pain (*P*_corrected_=1.14×10^–4^; OR_corrected_=1.22, 95% CI 1.11-1.36), and neck or shoulder pain (*P*_corrected_=6.99×10^–4^; OR_corrected_=1.19, 95% CI 1.08-1.31). Length of mobile phone use significantly increased the risk of MCP (*P*_corrected_=1.04×10^–4^; OR_corrected_=1.39, 95% CI 1.18-1.64), headaches (*P*_corrected_=.03; OR_corrected_=1.34, 95% CI 1.04-1.74), and neck or shoulder pain (*P*_corrected_=.01; OR_corrected_=1.55, 95% CI 1.1-2.17). In contrast, time spent using computer was negatively associated with the risk of MCP (*P*_corrected_=1.65×10^–3^; OR_corrected_=0.81, 95% CI 0.71-0.92), back pain (*P*_corrected_=4.28×10^–3^; OR_corrected_=0.83, 95% CI 0.73-0.94), and knee pain (*P*_corrected_=3.48×10^–3^; OR_corrected_=0.83, 95% CI 0.73-0.94). Overall, the MRlap-corrected causal estimates were consistent in direction and statistical significance with the primary IVW results in Multimedia Appendix 6, supporting the robustness of the findings against biases induced by sample overlap. Leave-one-out analyses in Multimedia Appendix 7 showed no outlying SNPs.

In the reverse MR analysis, MCP was used as the exposure, and different types of screen time were treated as outcomes. The F-statistics of IVs of MCP in Multimedia Appendix 5 are all greater than 10, indicating that the IVs are relatively strong. The results in Multimedia Appendix 8 indicated that MCP was positively associated with time spent watching television (*P*=4.8×10^–7^; OR 1.27, 95% CI 1.16-1.4) and length of mobile phone use (*P*=3.38×10^–5^; OR 1.29, 95% CI 1.14-1.45), while the association with time spent using computer (*P*=.61; OR 0.97, 95% CI 0.87-1.09) was not statistically significant. For other exposures, the analysis could not be conducted due to the limited number of available IVs or F-statistics below the conventional threshold of 10.

### Independent Effect of Screen Time on Chronic Pain

Multimedia Appendix 9 showed that the causal effects of screen time on chronic pain based on IVW MVMR. The conditional F-statistics for both time spent using computer and length of mobile phone use is less than 10 in the MVMR results.

### Discovery of Screen Time and Chronic Pain Genes Based on SMR Analysis

As the main analysis result, SMR was corrected by FDR and combined with the HEIDI test to screen for overlapping genes between exposure and outcome in 8 eQTL summary data. Multimedia Appendices 10 and 11 provided the full results, including the exact *P*_FDR_ and *P*_HEIDI_ values for all tested genes after FDR correction. A total of 72 overlapping genes related to exposure and outcome were identified in Multimedia Appendix 12, and the results showed that these overlapping genes originated from 3 exposures and MCP: 40 genes for time spent watching television and MCP, 26 genes for time spent using computer and MCP, and 6 genes for length of mobile phone use and MCP. In the SMR analysis, the Adipose Subcutaneous tissue had the most genes, while only one gene was found in the Brain Spinal cord cervical c-1.

### Genetic Findings of TWAS-Based Screen Time and Chronic Pain

After Bonferroni correction, we examined genome-wide significant associations in Multimedia Appendices 13 and 14, followed by joint and conditional tests. Multimedia Appendices 15 and 16 provided the results of the jointly significant genes. Then we identified 28 genes with overlapping exposures and outcomes in Table 1. No genes overlapping with the exposure were found in the hip pain for ≥3 months GWAS dataset.

**Table 1:** Transcriptome-wide significant genes identified by TWAS^a^ in screen time and chronic pain.

Trait	Cross-tissue expression	Gene (chromosome)
Time spent watching television – back pain for ≥3 months	sCCA1	*PMS2P3* (7)
Time spent watching television – MCP^b^	sCCA2	*FUBP1* (1) *WDR47* (1) *PLEKHO1* (1) *FAM172A* (5)
Time spent watching television – MCP	sCCA3	*SUSD3* (9) *RPL35* (9) *RELA* (11) *RMC1* (18) *SYPL2* (1) *SF3B4* (1) *ARPC5L* (9) *PTPDC1* (9) *FAM53B* (10)
Time spent watching television – knee pain for ≥3 months	sCCA2	*GATC* (12)
Length of mobile phone use – neck or shoulder pain for ≥3 months	sCCA3	*RBM42* (19)
Length of mobile phone use – MCP	sCCA3	*CSTPP1* (11) *TM9SF4* (20)
Time spent using computer – MCP	sCCA2	*WDR47* (1) *RFTN2* (2) *NMT1* (17) *RMC1* (18) *FASTKD5* (20) *TM9SF4* (20)
Time spent using computer – MCP	sCCA3	*SYPL2* (1) *CEP170* (1) *SCOC-AS1* (4)
Time spent using computer – headaches for ≥3 months	sCCA2	*SHMT2* (12)

^a^TWAS: transcriptome-wide association study.

^b^MCP: multisite chronic pain.

**Figure 3:**
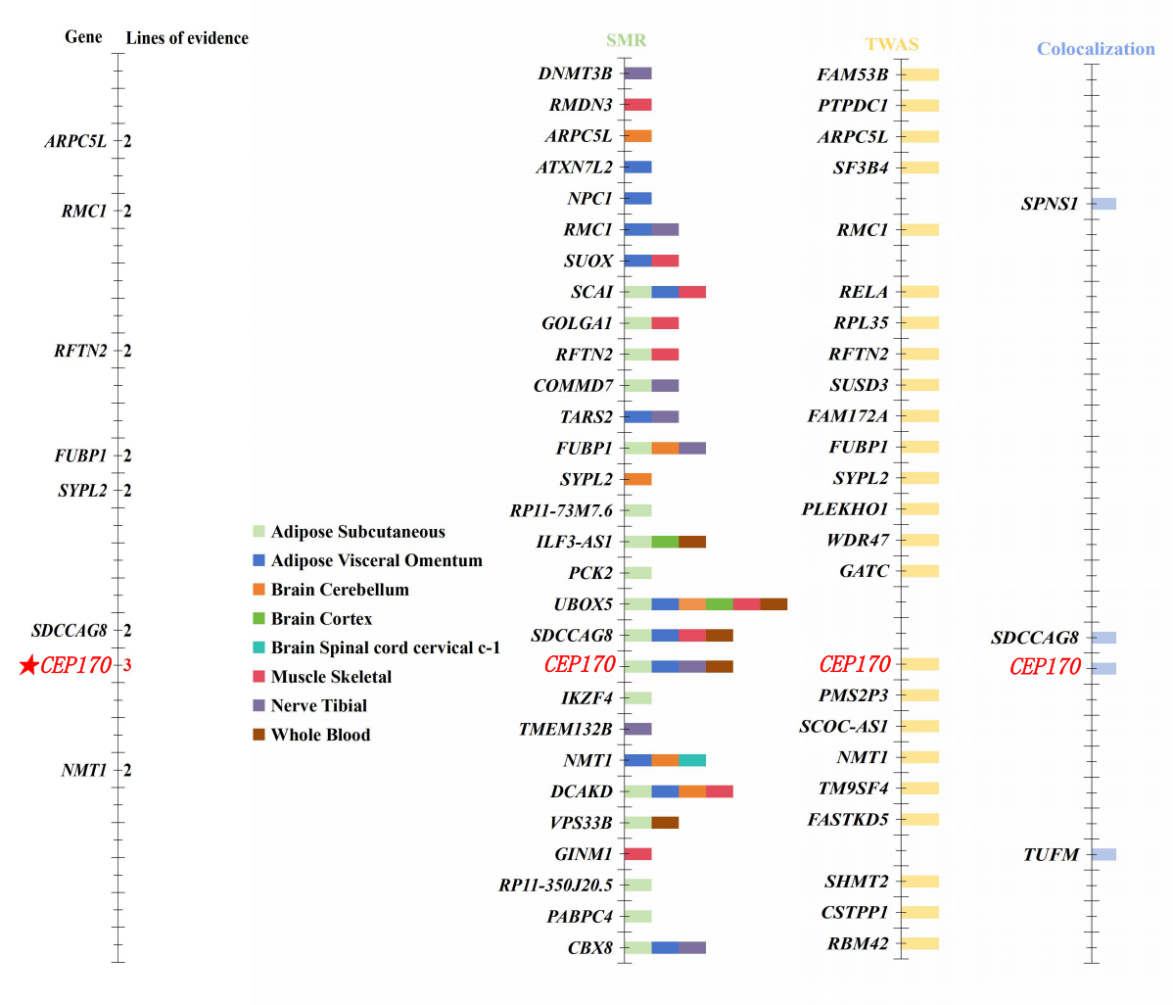
Results supported by genetic evidence. SMR: summary data–based Mendelian randomization analysis; TWAS: transcriptome-wide association study.

By combining the results of SMR and TWAS analysis, we found that 7 overlapping genes (*SYPL2*, *RMC1*, *FUBP1*, *ARPC5L*, *RFTN2*, *NMT1*, and *CEP170*) were identified between exposures (time spent watching television and time spent using computer) and outcome (MCP) in Multimedia Appendix 17 and Figure 3. In addition, we found that *SYPL2* gene expression was associated with both time spent watching television and time spent using computer.

In Figure 3, the panel on the far left shows genes supported by at least 2 lines of evidence. A total of 3 lines of evidence were used to support genes, with each column representing one type of supporting evidence. The SMR evidence shows different colors according to 8 different eQTL summary data. TWAS evidence is shown in light yellow and colocalization evidence is shown in light blue. *CEP170* was supported by all analyses. *ARPC5L*, *RMC1*, *RFTN2*, *FUBP1*, *SYPL2*, *SDCCAG8*, and *NMT1* were supported by 2 lines of evidence.

### Colocalization Analysis of Shared Genetic Variation

Colocalization analysis identified multiple genes with moderate or strong support for colocalization signals in the shared genetic variation between screen time and chronic pain in Multimedia Appendix 18. The results showed that 4 genes suggest a moderate support for colocalization (*SPNS1*, *TUFM*, *SDCCAG8*, and *CEP170*). Multimedia Appendix 19 showed that the 4 genes are overlapped between screen time and chronic pain.

### Gene Discovery

As shown in Figure 3, we presented the distribution of significant genes in each analysis method, and the results showed that the *CEP170* gene stood out in the association analysis of “Time spent using computer” and MCP, receiving triple support from SMR, TWAS, and colocalization analysis (PPH_4_>.75). The signaling distribution of this gene involved 4 eQTL tissues: Adipose Subcutaneous, Adipose Visceral Omentum, Nerve Tibial, and Whole Blood.

## Discussion

### Principal Findings

This study delves into potential phenotypic associations and underlying genetic connections between screen time and chronic pain. The results show that screen time is associated with chronic pain. In the 2-sample MR results, time spent watching television and length of mobile phone use were significantly and positively associated with chronic pain, including MCP, back pain, knee pain, neck or shoulder pain, hip pain, and headaches. In contrast, time spent using computer inversely was negatively associated with MCP, back pain, and knee pain. It is particularly important to note that we still list the significant IVW results of exposure to the length of mobile phone use. Even though the effect value directions of the IVW and MR-Egger results are not consistent, it is consistent with the direction of our main analytical method in other methods. The statistical power of MR-Egger decreases for fewer IVs [27]. Therefore, we still take the results of IVW as the standard for the length of mobile phone use. In the reverse MR results, we found that there is a bidirectional positive correlation among time spent watching television, length of mobile phone use, and MCP. In the MVMR analysis, not all the conditional F-statistics were >10, suggesting the presence of weak instruments. Therefore, our interpretations were based on the 2-sample MR results. However, the inverse association between time spent using computer and MCP should be interpreted cautiously, as it stems from the 2-sample MR results. The lack of robust MVMR findings means we cannot rule out the potential influence of confounding factors. In addition, by integrating SMR, TWAS, and colocalization analysis, we identified an overlapping gene *CEP170* associated with time spent using computer and MCP. These findings highlight the important role of screen time in the development of chronic pain.

### Differential Associations Between Screen Time and Chronic Pain

Previous studies have shown a positive correlation between mobile phone use and neck pain [39], and a strong correlation between watching television and back pain [40], which is consistent with our research findings. In contrast, our research found that time spent using computer may have a protective effect on certain types of chronic pain, which differs from some studies that concluded, “longer computer time increases the risk of multi-site pain” [8,39]. This difference may be due to the study population, with earlier studies focusing on adolescents, while our analysis was conducted on adults. Due to continued musculoskeletal development, adolescents may be more susceptible to the negative physical effects of sedentary behavior.

Furthermore, 2 potential explanations may underlie the differential associations between time spent using computer and chronic pain, although there is no direct evidence available at present. One possible explanation is that the perception of pain is influenced by the allocation of cognitive resources; that is, when attention is occupied by other highly attractive stimuli, the perception of pain may be reduced [41,42]. According to the characteristics of the UK Biobank population (aged 40-69 years during 2006-2010) in this study [43], the use of mobile phones and televisions was often associated with activities that had relatively low cognitive requirements and less attention input at that time. In contrast, even leisure-time computer use generally involved more cognitively demanding activities among middle-aged adults, such as reading, writing, online communication, or other interactive tasks. Research indicates that pain is less likely to enter conscious awareness when cognitive resources are occupied by goal-directed information maintained in working memory and when sustained attention is devoted to the task [44]. This indicates that computer tasks with high cognitive load may potentially reduce the pain experience through distraction. Moreover, it is also important to consider that individuals who frequently use computers tend to have higher educational attainment, higher income, and better overall health [45]. Given that individuals in nonmanual occupations tend to have a lower likelihood of experiencing chronic pain compared with those in manual labor occupations [46,47], part of the observed lower risk associated with time spent using computer may therefore reflect underlying socioeconomic or occupational factors rather than a sole reflection of the nature of computer-based activities themselves. This may explain why length of mobile phone use and time spent watching television were associated with a higher risk of pain, while time spent using computer showed a protective effect in this study.

Another possible explanation is that different types of screen time may affect pain perception by engaging the brain’s reward system to varying degrees. Dopamine is involved in pleasure, reward, and incentive behavior [48]. Although mobile phone and television use may induce pleasure, the limited interactivity prevents them from triggering dopamine release to the same extent as engaging in more goal-directed and cognitively engaging computer activities. Successfully completing such goals may elicit a sense of accomplishment. The reward system may be activated, promoting dopamine release, which has been proven to have analgesic effects [49,50]. Moreover, bidirectional association analysis suggests that MCP may influence the choice of screen behavior. People with MCP tend to increase passive and low-effort screen behaviors (such as watching television or casually browsing their mobile phones). This behavioral adaptation may contribute to a pattern of bidirectional reinforcement, where an increase in passive activity further restricts physical movement and may exacerbate pain. However, we emphasize that these explanations are speculative, as direct evidence is currently lacking.

### The Potential Mechanisms by Which CEP170 Contributes to Screen Time and Chronic Pain

Leveraging multiomics approaches, we identified *CEP170*, whose cis-regulated expression may contribute to the biological mechanisms between time spent using computer and MCP. In SMR results, the *CEP170* gene expression level was positively correlated with MCP but negatively correlated with time spent using computer. This aligns with our MR results, where time spent using computer was associated with a lower risk of MCP. One possible explanation is that *CEP170* may influence behavioral patterns related to screen time, where individuals with higher gene expression tend to spend less time on the computer, which in turn is associated with increased MCP risk. Although its colocalization results suggest a moderate support for colocalization, *CEP170* was detected in all analytical methods.

*CEP170* is located at the centrosome and spindle microtubules and participates in microtubule organization and assembly [51,52]. Additionally, *CEP170* plays an important role in supporting ciliary homeostasis [53]. Cilia may potentially play a central role in cell signaling, and recent animal experiments have further revealed that cilia have key regulatory functions in controlling mechanical nociceptive thresholds and inflammatory and neuropathic pain [54]. The SMR results show that *CEP170* exhibited significant signals in multiple eQTL tissues, including Adipose Subcutaneous, Adipose Visceral Omentum, Nerve Tibial, and Whole Blood. This indicates that *CEP170* may be expressed in these tissues and may contribute to the biological mechanisms behind the observed associations. A large UK Biobank study revealed that abdominal adipose tissue (including visceral fat and subcutaneous fat) was associated with chronic musculoskeletal pain. It suggested that excessive and ectopic fat depositions may be involved in the pathogenesis of multisite and widespread chronic musculoskeletal pain [55]. This is likely attributable to the chronic low-grade inflammation driven by accumulated visceral fat, which can sensitize peripheral nerves and promote MCP [56,57]. In line with this, our SMR analysis found that *CEP170* expression in subcutaneous and visceral adipose tissue is associated with MCP. Given the established importance of microtubules in lipid metabolic homeostasis [58,59], we hypothesize that *CEP170* may potentially protect against MCP by enhancing microtubule stability in adipose tissue. This mechanism might potentially constrain adipose-derived inflammatory signaling, thereby possibly alleviating peripheral nerve sensitization and lowering MCP risk. However, the current results only provide preliminary genetic clues. Direct links remain unclear and more functional studies, especially tissue-specific experiments, are needed in the future to clarify the potential contribution of *CEP170* to pain pathways.

### Strengths and Limitations

Our research has several advantages. First, we integrated a variety of analytical methods to duplicate our findings and strengthen the robustness of our results. Second, we leveraged large-scale GWAS data from the UK Biobank, ensuring a robust analysis with broad generalizability. Third, by identifying overlapping genes and potential biological pathways, our study provides new genetic insights into the association between screen time and chronic pain, which, despite limited evidence, may inform future research in this field. Finally, our study considered different types of screen time separately, which allows for a more nuanced understanding of their different effects on chronic pain. These findings may serve as a useful reference for subsequent studies on the genetic and epidemiological links between screen time and chronic pain.

Although this study provides new insights, there are still some limitations. First, using data from the same sources in multiple analyses may introduce the bias of the winner’s curse, potentially inflating effect sizes or significance levels. Replication in fully independent datasets is necessary to confirm the discovery. Second, our analyses were restricted to European populations, which may limit generalizability to other populations with different screen use habits and pain reporting. Third, the lack of detailed data on screen use posture, activity type, and behavior patterns may limit the depth of causal reasoning. Future studies could incorporate longitudinal and behavioral data to better clarify the mechanisms and causal pathways. Fourth, as both exposures and outcomes are self-reported, recall and measurement bias may lead to misclassification and reduce precision. Self-reported pain may not capture clinical heterogeneity, so future studies using clinically validated phenotypes are needed. Fifth, due to the weak strength of IVs, we were unable to adequately control for crucial potential confounding factors, such as socioeconomic status and educational attainment. Consequently, the observed protective association between “time spent using computer” and chronic pain might be partially attributable to residual confounding by socioeconomic status and related factors. Sixth, reverse MR could not be performed for the outcomes of back pain, knee pain, neck or shoulder pain, headaches, and hip pain due to limited IVs, limiting the assessment of reverse causality. In addition, the smaller sample size for hip pain may have limited statistical power, contributing to borderline significance in the 2-sample MR analysis and precluding reverse MR for this phenotype. Seventh, environmental exposure, such as air pollution, was not taken into account in this analysis. Particulate matter can affect inflammatory and stress-related biomarkers involved in chronic pain [60-62]. Future studies should integrate genetic and environmental data to clarify these relationships. Eighth, the colocalization signal for *CEP170* is not the strongest, and direct evidence is lacking. Therefore, its role remains speculative and warrants further investigation. Finally, this study lacks validation in large surveys or independent cohorts, highlighting the need to combine genetic and epidemiological studies in future research.

### Conclusions

This study provides evidence that reveals an association between screen time and chronic pain. We found the *CEP170* gene might contribute to the shared biological mechanism between time spent using computer and MCP risk. Future studies should further validate this association and clarify the functional role of *CEP170* in the development of chronic pain.

## Appendix

Multimedia Appendix 1 Data source of the exposures and outcomes.

Multimedia Appendix 2 Characteristics of genetic instruments of Time spent watching television and their effect sizes with chronic pain.

Multimedia Appendix 3 Characteristics of genetic instruments of Time spent using computer and their effect sizes with chronic pain.

Multimedia Appendix 4 Characteristics of genetic instruments of Length of mobile phone use and their effect sizes with chronic pain.

Multimedia Appendix 5 Characteristics of genetic instruments of multisite chronic pain.

Multimedia Appendix 6 Putative causal effect of screen time on chronic pain from 2-sample Mendelian randomization analysis.

Multimedia Appendix 7 The leave-one-out plot of screen time on chronic pain.

Multimedia Appendix 8 Reverse Mendelian randomization analysis assessing the causal effect of chronic pain on screen time.

Multimedia Appendix 9 Causal effects of the screen time on chronic pain based on inverse variance weighted multivariable Mendelian randomization.

Multimedia Appendix 10 The top hit probes identified in summary data–based Mendelian randomization analysis after false discovery rate correction of screen time.

Multimedia Appendix 11 The top hit probes identified in summary data–based Mendelian randomization analysis after false discovery rate correction of chronic pain.

Multimedia Appendix 12 The genes with overlapping exposure and results were screened by PFDR and PHEIDI in summary data–based Mendelian randomization analysis.

Multimedia Appendix 13 Transcriptome-wide association study of screen time in three cross-tissue (sparse canonical correlation analysis) weights using Bonferroni correction and cross-tissue expression Genotype-Tissue Expression v8.

Multimedia Appendix 14 Transcriptome-wide association study of chronic pain in three cross-tissue (sparse canonical correlation analysis) weights using Bonferroni correction and cross-tissue expression Genotype-Tissue Expression v8.

Multimedia Appendix 15 The results of the jointly significant genes for screen time.

Multimedia Appendix 16 The results of the jointly significant genes for chronic pain.

Multimedia Appendix 17 Overlapping genes by both transcriptome-wide association study and summary data–based Mendelian randomization methods.

Multimedia Appendix 18 Results of the colocalization analysis for genes identified in the exposure and outcome traits.

Multimedia Appendix 19 Overlapping genes in colocalization.

## Abbreviations

eQTL: expression Quantitative Trait Locus
FDR: false discovery rate
GTEx: Genotype-Tissue Expression
GWAS: genome-wide association study
HEIDI: heterogeneity in dependent instruments
IEU: Integrative Epidemiology Unit
IV: instrumental variable
IVW: inverse variance weighted
LD: linkage disequilibrium
MAF: minor allele frequency
MCP: multisite chronic pain
MR: Mendelian randomization
MR-PRESSO: Mendelian Randomization Pleiotropy Residual Sum and Outlier
MVMR: multivariable Mendelian randomization
OR: odds ratio
PP: posterior probability
sCCA: sparse canonical correlation analysis
SMR: summary data–based Mendelian randomization
SNP: single-nucleotide polymorphism
TWAS: transcriptome-wide association study

## Data Availability

The code used in this study is available from the corresponding authors on reasonable request. The GWAS summary statistics data used in this study are available from IEU OpenGwas and publicly available GWAS. The eQTL summary data for GTEx used in the SMR analysis are available in the public database [63]. Precomputed sCCA weights for GTEx v8 used in this study are available in the public database [64]. The eQTL data used in the colocalization analysis are available from the eQTLGen Consortium [65].
