# Supplementary material for: Screen Time and Chronic Pain Health: Mendelian Randomization Study
Source: J Med Internet Res. 2026 Feb 9;28:e78233. doi: 10.2196/78233 (PMC12930148; doi:10.2196/78233)
Supplement: Multimedia Appendix 7 [file jmir_v28i1e78233_app7.docx]

**
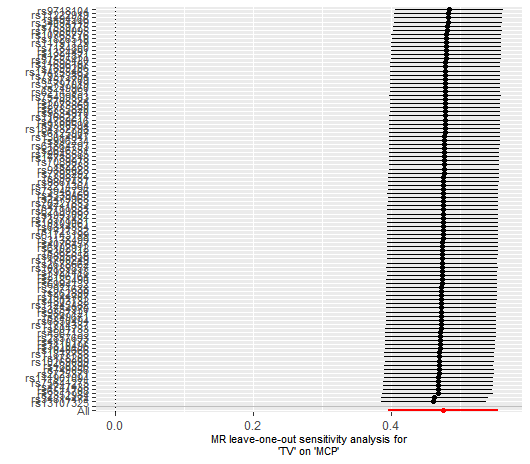

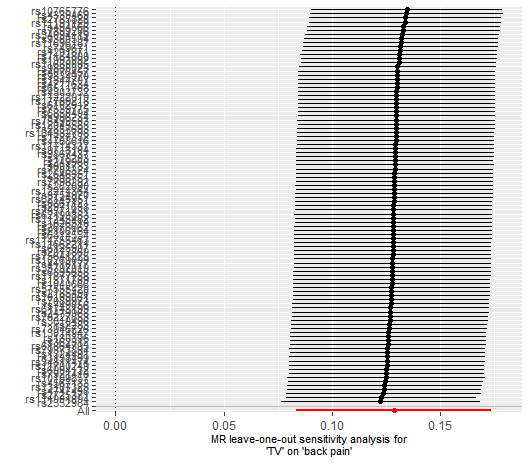

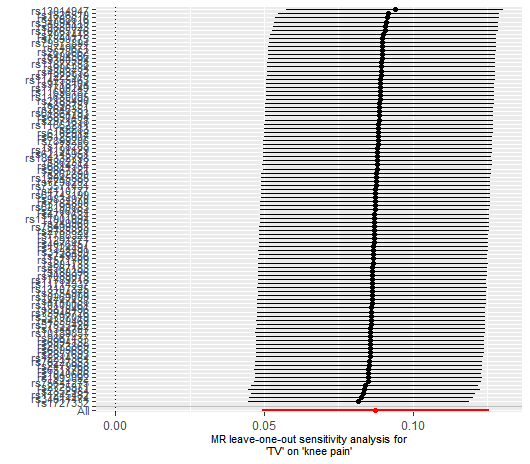
**

**
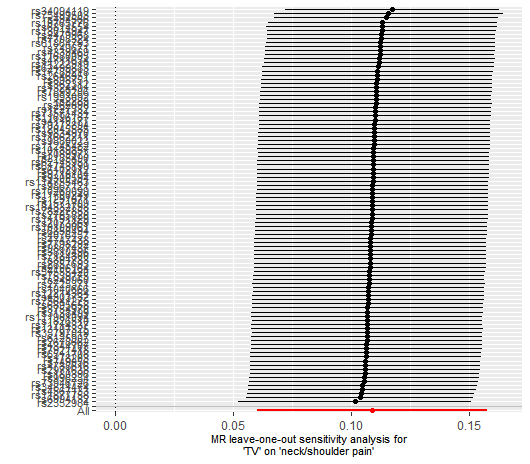

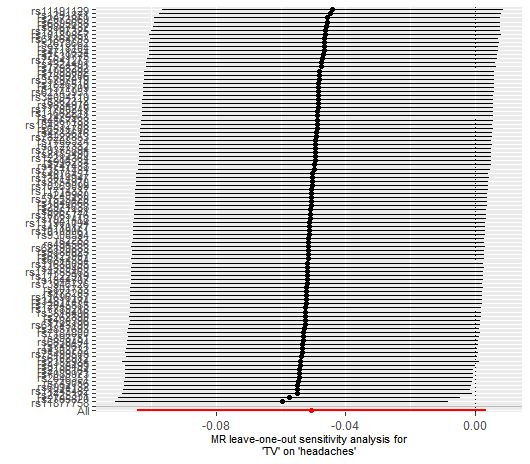

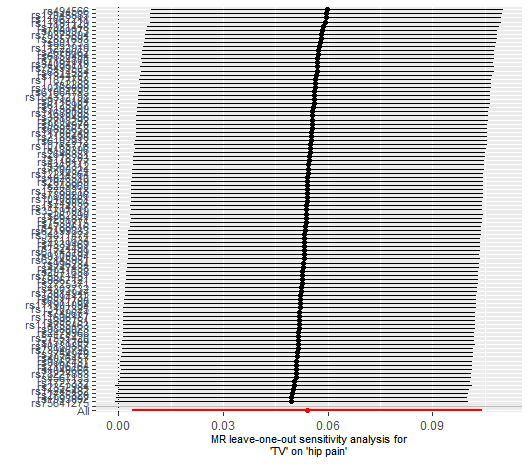
**

**
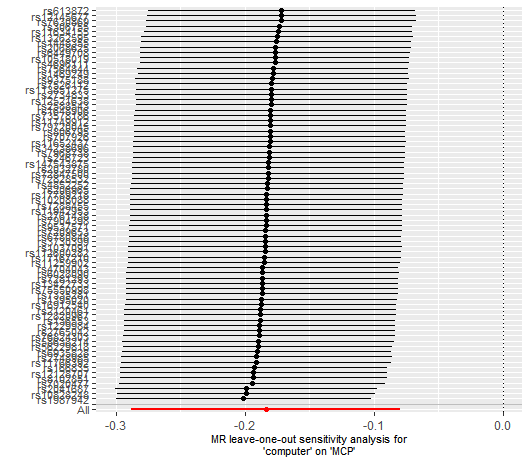

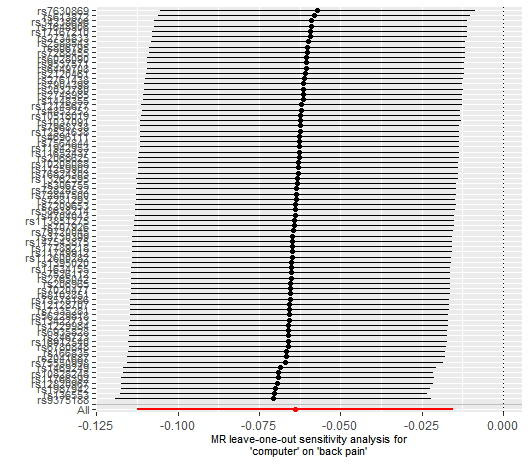

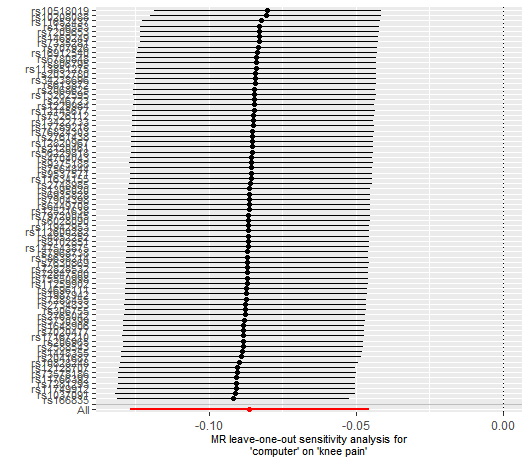
**

**
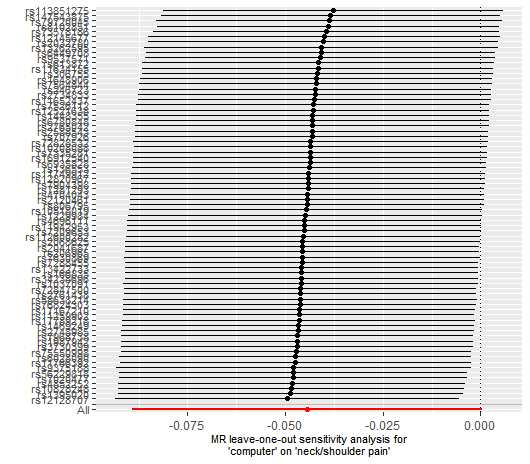

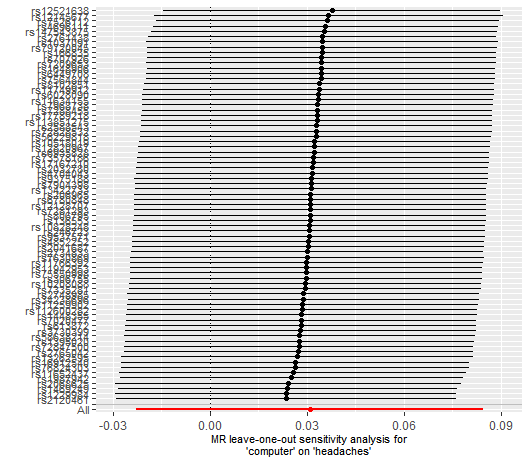

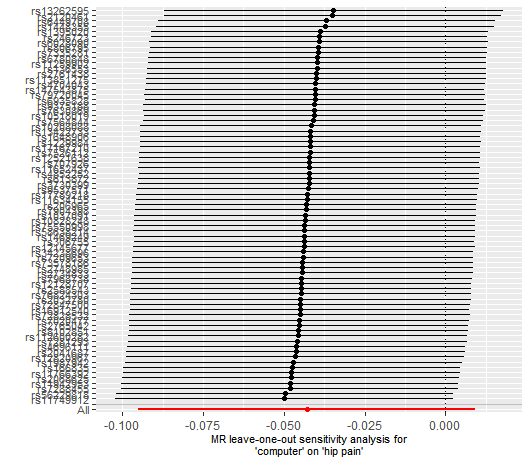
**

**
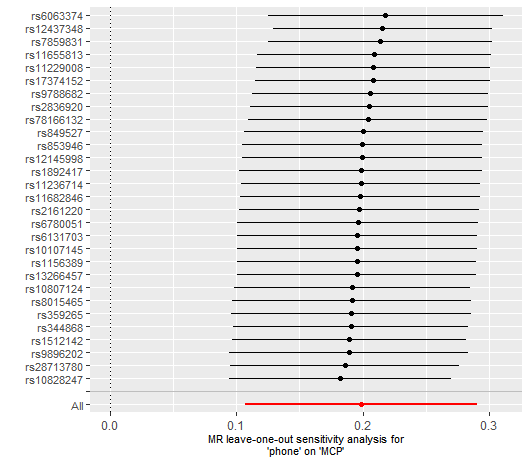

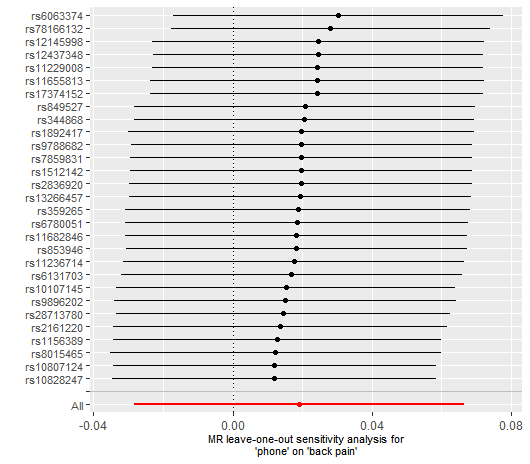

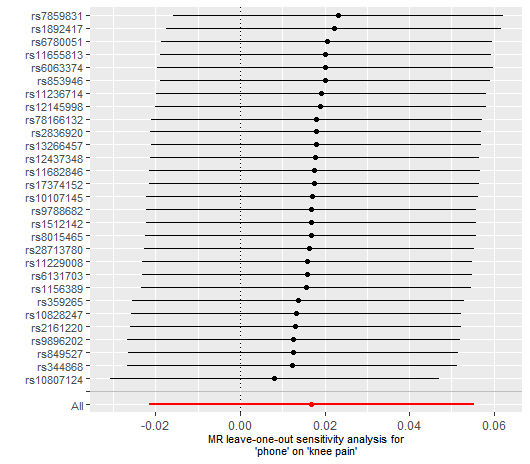
**

**
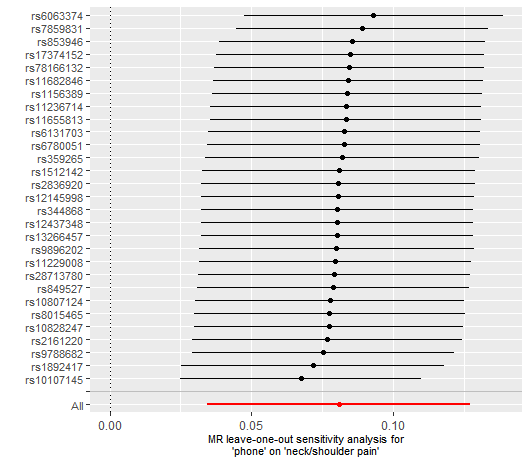

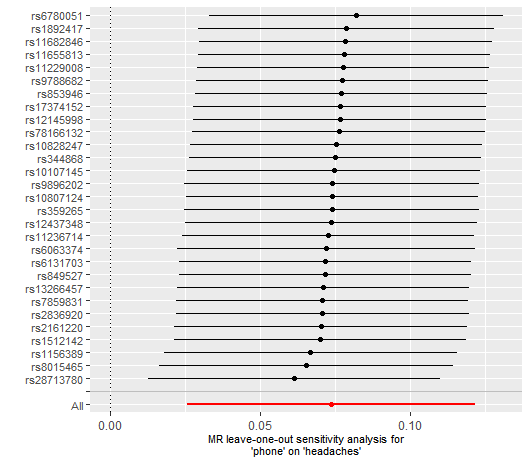

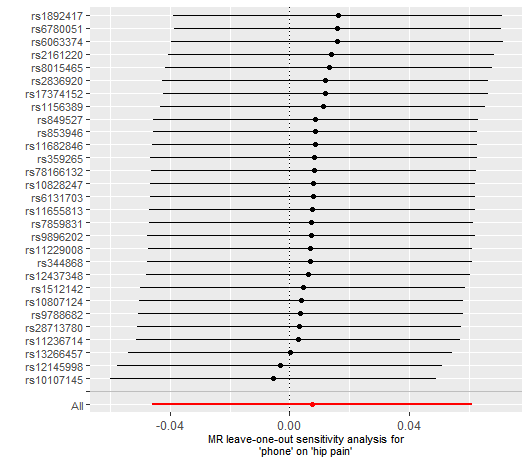
**

**Multimedia Appendix 7. The leave-one-out plot of screen time on chronic pain.**

MCP: multisite chronic pain; TV: television.
